# Well-Being and Perfectionism: Assessing the Mediational Role of Self-Compassion in Emerging Adults

**DOI:** 10.3390/ejihpe14050091

**Published:** 2024-05-15

**Authors:** Loredana Benedetto, Stefania Macidonio, Massimo Ingrassia

**Affiliations:** Department of Clinical and Experimental Medicine, University of Messina, 98125 Messina, Italy; stefaniamacidonio23@gmail.com

**Keywords:** psychological well-being, perfectionism, self-compassion, emerging adults

## Abstract

The study explores how different dimensions of perfectionism influence psychological well-being (PWB) in emerging adults. Literature has deepened the relationships between maladaptive perfectionism (e.g., excessive self-criticism, perceived discrepancy from the standards) and low PWB. Less is known about whether and how adaptive perfectionism (e.g., pursuing personal standards) relates to PWB. Secondly, the study has investigated whether self-compassion (i.e., self-benevolence, seeing personal imperfections as a common condition) may mediate the relationships between adaptive/maladaptive perfectionism and PWB. Participants (N = 217, 18–35 y. o.) completed self-report questionnaires measuring: adaptive/maladaptive perfectionism (Almost Perfect Scale-Revised, APS-R: high standards and order/discrepancy, respectively), PWB, and self-compassion (SCS). Adaptive perfectionism was associated with PWB, particularly a higher presence of purpose in life, environmental mastery, self-acceptance, and personal growth. Conversely, discrepancy resulted in the most robust predictor of low PWB (β = −0.68), followed by high standards with a positive direction (β = 0.23; Rc^2^ = 0.514, *p* < 0.001). A strong negative association emerged between discrepancy and SCS (r = −0.67, *p* < 0.001). A mediation analysis shows that self-compassion has an indirect effect, reducing the strength of the relationship between discrepancy and low PWB. Results suggest focusing on self-compassion as a buffer factor that reduces the negative impact of maladaptive perfectionism on psychological well-being. Implications for education and health psychology are discussed.

## 1. Introduction

Going beyond a one-dimensional conception of perfectionism and its role in many health problems (i.e., depression and eating disorders; [1]), 30 years of research have contributed to the shift toward a multifaceted view of perfectionism in which healthy and unhealthy components coexist [2]. Perfectionism is a multidimensional personality trait characterized by tendencies to strive for excellence, to be overly critical of one’s performance when the results do not meet expectations, and to perceive high levels of distress from mistakes [3,4,5]. 

The coexistence of positive and negative aspects of perfectionism was proposed among the first ones by Hamachek [6], who distinguishes between normal and neurotic forms of perfectionistic traits. A key difference is that adaptive perfectionists set realistic goals for themselves, and experience satisfaction with their efforts and accomplishments, but they are also flexible enough to tolerate occasional mistakes. Conversely, neurotic perfectionists are characterized by setting unrealistic performance standards and feeling worry and dissatisfaction with their own performances. Neurotic perfectionism may be driven by the fear of failure, rather than the desire to achieve, and may lead to negative outcomes such as procrastination, indecisiveness, and distress [7]. 

The distinction between normal/adaptive and neurotic/maladaptive perfectionism is supported by subsequent studies. In their review, Stoeber and Otto [8] outlined two higher-order dimensions that emerge across studies: Perfectionistic strivings (setting high expectations for performance) and perfectionistic concerns (self-criticalness regarding performance). Perfectionistic striving describes people’s tendency to establish high goals and expectations for themselves (e.g., intrinsically motivated, or self-oriented perfectionism), and to search for order and organization. These personal tendencies are not necessarily negative, and they are associated with conscientiousness, positive affect, and problem-focused coping [9]. Conversely, perfectionistic concerns have been conceptualized in many as the maladaptive face of perfectionism: They include individuals’ tendency to believe that their significant others expect high standards from them (e.g., socially prescribed perfectionism), over-criticism of their own performances, and doubts about their ability to achieve the established standards [3,10]. Particularly, expectations of excellence and criticism from significant adults (mainly, parents and teachers) may be a developmental antecedent of perfectionistic tendencies [11]. Slaney and colleagues [12] introduce discrepancy as a key dimension for maladaptive perfectionism, whereas high standards and order are viewed as the adaptive faces of perfectionism. The discrepancy is focused on the perception of the gap between one’s standards and actual performance, excessive self-criticism for one’s own performance, and negative feelings due to the inability to achieve true perfection (i.e., “Doing my best never seems to be enough”). Assuming this multifaceted view of perfectionism, many studies have focused on the impact of maladaptive perfectionism on poor psychological functioning. Studies report that maladaptive perfectionism is generally associated with higher stress, anxiety, avoidant coping, low self-esteem and academic self-efficacy, eating disorders, depression, and suicide (for reviews, see [13,14]). Recently, perfectionism was explored in relation to distress and fear linked to the COVID-19 pandemic [15]. However, compared to the large body of literature on negative outcomes, the role of perfectionism in healthy functioning remains a research field that has been partially neglected.

Previous studies have demonstrated the link between adaptive perfectionism and indicators of healthy functioning, including challenge appraisals and effective coping [9], self-efficacy and academic achievement [16,17], happiness [18,19], and life satisfaction [20]. In addition, other studies suggest that some mediating variables can intervene in the relationship between perfectionism and healthy outcomes, such as self-esteem [21] and social problem-solving [22], buffering the impact of maladaptive perfectionism on depressive symptoms. Particularly, self-compassion recently emerged as a moderating variable that alters the association between maladaptive perfectionism and health problems, including distress, anxiety, and depression [23].

According to Neff [24], self-compassion is defined as “a healthy attitude toward oneself” during life difficulties, that is, a mindset characterized by feelings of self-benevolence and absence of criticism when one suffers, fails, or feels inadequate. Self-compassion has three basic interacting components: Self-kindness versus self-judgment (being warm and kind to oneself instead of blaming for their own sufferings and negative feelings), common humanity versus isolation (recognizing that imperfections and failures are inevitable and that everyone can experience them as part of the human condition), and mindfulness versus over-identification (being aware and uncritical towards one’s painful thoughts and feelings, without repressing nor exaggerating them). Studies confirm that self-compassion weakens the influence of maladaptive perfectionism on negative affect and depressive symptoms [25,26]. Conversely, low self-compassion levels predict general distress and anxious symptoms in individuals with higher self-critical perfectionism traits [27]. Alongside these empirical data supporting the role of self-compassion as a buffer variable for the negative impact of maladaptive perfectionism, few studies have investigated both the constructs of multidimensional perfectionism and self-compassion in relation to well-being indicators.

Moving from these premises, the current study aimed to expand on previous findings on multidimensional perfectionism, self-compassion, and psychological well-being in emerging adults. First, we assumed Ryff’s perspective on psychological well-being was intended as the degree to which an individual experiences self-realization and positive functioning in his/her own life circumstances [28]. This eudaimonic perspective of psychological well-being differs from a hedonic perspective or subjective well-being (i.e., happiness or a pleasant life). Psychological well-being combines feeling good and positive affective states (i.e., self-acceptance, positive social relations) with the individual’s perception of engagement and self-realization in activities that are congruent with personal values. In accordance with Ryff’s theoretical model, self-acceptance, autonomy, personal growth, environmental mastery, positive relations, and purpose in life are the core dimensions of psychological well-being [29]. This perspective describes positive human functioning as a process of self-realization and development (personal growth) in which the person recognizes his/her capabilities (self-acceptance), makes decisions, and pursues goals independently from external influences (autonomy). Psychological well-being comes from cultivating positive social relationships, not just from the perception of personal competence (environmental mastery) in various life circumstances, but mainly from experiences that realize one’s values and lead to increased meaning (purpose in life). These dimensions emphasize people’s ability to achieve what they want out of life, to improve, and to cope with challenges; therefore, they could be linked with the adaptive expressions of perfectionism [19]. In fact, adaptive perfectionists set elevated standards, with organizations reporting higher levels of perceived satisfaction, self-esteem, and meaning in life than maladaptive perfectionists [30]. Several studies confirm the relationships between different faces of perfectionism and the dimensions of eudaimonic well-being. Adaptive perfectionism was positively associated with environmental mastery and purpose in life as indices of psychological well-being [31]. Conversely, maladaptive perfectionism was found to be associated with low psychological well-being (precisely, autonomy, environmental mastery, and purpose in life; [32]). In accordance with these premises, the hypothesis we assumed is the following:

**Hypothesis 1** **(H1).** *The indices of psychological well-being [29] are negatively associated with discrepancy and positively with adaptive perfectionism (high standards/order), according to the perfectionism dimension proposed by Slaney and colleagues [12].*

Secondly, the link between adaptive/maladaptive perfectionism and self-compassion was explored. Previous studies have reported mixed results, which only partially confirm this relationship. Specifically, in most studies [24,26,33,34] maladaptive perfectionism showed moderate-to-large associations with lower self-compassion levels. Maladaptive perfectionism drives the individual to focus mainly on shortcomings and performances that are perceived to be below expectations. This excessive self-criticism is the opposite of a self-compassionate attitude, which makes people tolerant of imperfection and benevolent towards their own failures. Therefore, the hypothesis to test is that:

**Hypothesis 2** **(H2).** *Maladaptive perfectionism (discrepancy) has moderate-to-large associations with lower levels of self-compassion.*

Conversely, the results of studies partially confirm the link between self-compassion and the adaptive dimensions of perfectionism. In some studies, a small positive correlation was found [33,35], but in others, no significant association emerged [24]. Therefore, by posing an exploratory hypothesis, we expect:

**Hypothesis 3** **(H3).** *Positive associations between the adaptive faces of perfectionism and self-compassion, exhibiting different strengths (weak-to-null) with the different dimensions of positive perfectionism (i.e., high standards and order).*

Finally, the current study investigates whether self-compassion may mediate the relationships between adaptive/maladaptive dimensions of perfectionism and psychological well-being. The role of self-compassion in mitigating the unhealthy impact of maladaptive perfectionism is well documented in the literature. Particularly, Mehr and Adams [25], among others, found that self-compassion buffered the impact of maladaptive perfectionism and depressive symptoms. Similarly, Wei and colleagues [33] showed that adaptive perfectionism and self-compassion negatively predicted depression levels; moreover, self-compassion moderated the impact of maladaptive perfectionism on depression levels. Conversely, studies on health outcomes are scarce. Şahin [36] observed that self-compassion fully mediates the relationship between maladaptive perfectionism and life satisfaction, but no significant relationship between adaptive perfectionism and life satisfaction emerged. In the current study, the hypothesis to test is:

**Hypothesis 4** **(H4).** *Self-compassion can intervene as a buffer variable between maladaptive perfectionism and psychological well-being.*

## 2. Materials and Methods

### 2.1. Participants 

Data were collected from a community sample (N = 217) of young adults (18–35 y. o.; M = 23.7, SD = 3.3), mainly females (N = 150, 69.1%). Participants declared to be college students (64.6%), employed (25.4%), or unemployed (8.8%), while a small number did not report an occupation (1.2%). Almost all respondents were unmarried (90%), married (4%), or cohabiting (6%).

### 2.2. Procedure

The participants, all volunteers, were recruited through social networks using Google Forms. The online survey platform started with a brief presentation of the study’s aim, followed by the consent form. After giving their consent, participants were directed to the online questionnaire. The answers of each participant were aggregated into the database and stored without any personal identification code. The requested demographics were age, gender, marital status, and profession. Informed consent was obtained from all subjects involved in the study.

### 2.3. Measures

The Almost Perfect Scale-Revised (APS-R; [12]) in the 20-item Italian adaptation [37] was used as a measure for the adaptive/maladaptive tendencies of perfectionism. The dimensions that are assumed to be adaptive are order (4 items, e.g., “I always like to be organized and disciplined”) and high standards (6 items, e.g., “I set very high standards for myself”). The discrepancy sub-scale (10 items) measures maladaptive aspects of perfectionism, such as “I often feel frustrated because I can’t meet my goals”. Responses are rated on a 7-point Likert scale from 1 (strongly disagree) to 7 (strongly agree), with higher scores indicating stronger perfectionistic tendencies. The internal consistency (Cronbach’s α test) of the Italian questionnaire was adequate in the three dimensions: high standards 0.71, order 0.81, and discrepancy 0.91. 

The Self-Compassion Scale (SCS, [38]; It. ad. [39]) is a self-report questionnaire (26 items) measuring the components of self-compassion. The sub-scales with positive direction are self-kindness (e.g., “I try to be loving towards myself when I’m feeling emotional pain”), common humanity (“I try to see my failings as part of the human condition”), and mindfulness (“When I’m feeling down, I try to approach my feelings with curiosity and openness”). The negative-reversed sub-scales are self-judgment (e.g., “I’m disapproving and judgmental about my own flaws and inadequacies”), isolation (“When I think about my inadequacies it tends to make me feel more separate and cut off from the rest of the world”), and over-identification (“When I’m feeling down I tend to obsess and fixate on everything that’s wrong”). Respondent rates each item on a 5-point Likert scale from 1 (almost never) to 5 (almost always). Mean scores on the six sub-scales are then averaged after reversing the scores of items with a negative direction. The overall self-compassion score ranges from 26 to 130, with higher scores indicating higher levels of self-compassion. In the present study, the total SCS had excellent internal consistency (α = 0.92).

The Psychological Well-Being Scales (PWB) by Ryff and Keyes ([40]; It. ad. [41]) is a self-report questionnaire assessing six areas: Autonomy (e.g., “I have confidence in my own opinions, even if they are different from the way most other people think”), self-acceptance (“When I look at the story of my life, I am pleased with how things have turned out”), environmental mastery (“In general, I feel I am in charge of the situation in which I live”), personal growth (“I think it is important to have new experiences that challenge how you think about yourself and the world”), purpose in life (“I am an active person in carrying out the plans I set for myself”), and positive relations (“Most people see me as loving and affectionate”). Responses are expressed on a 6-point Likert scale from 1 (I do not agree) to 6 (completely agree), with high scores indicating perceived satisfaction and mastery in that area of personal life. In this study, the internal consistency of the 54-item questionnaire resulted in an excellent total scale (α = 0.95) and ranged from 0.53 (environmental mastery) to 0.89 (self-acceptance) for subscales. 

## 3. Results

### 3.1. Statistical Analysis

Data were processed using IBM SPSS Statistics for Windows 19.0. The first step was to calculate descriptive statistics (M, SD) for study measures: Dimensions of perfectionism (APS-R sub-scales), self-compassion (SCS sub-scales), and psychological well-being (PWB sub-scales). Secondly, a correlational analysis (Pearson’s r) was performed to explore the relationships between study measures. Then, a linear stepwise regression analysis was calculated. Perfectionism dimensions (APS-R subscales) and self-compassion (SCS total score) were used to test factors predicting psychological well-being levels (PWB total score). Finally, a mediational analysis was calculated to test the direct and indirect effects of the relationship between maladaptive perfectionism and psychological well-being as mediated by self-compassion.

### 3.2. Preliminary Analysis

Descriptive statistics and bivariate Pearson’s correlations are presented in Table 1. Results show moderate to strong negative correlations (*p* < 0.01) between discrepancy (maladaptive perfectionism) and well-being dimensions. Regardless of adaptive perfectionism, high standards showed weak to moderate positive correlations with well-being sub-scales, except for positive relations and autonomy, where no significant associations emerged. Order has a weak but significant positive association with the environmental mastery sub-scale (*p* < 0.01). With respect to self-compassion, no association resulted in high standards, whereas a strong negative association emerged with discrepancy (*p* < 0.001). Finally, well-being has significant correlations (*p* < 0.05 and 0.01) with self-compassion dimensions, particularly with subscales with a positive direction: self-kindness, common humanity, and mindfulness have weak to strong Pearson’s correlations with all dimensions of psychological well-being.

### 3.3. Predictors of Psychological Well-Being

The regression analysis (Table 2) revealed a model with two factors explaining 51% of variability (R_c_^2^ = 0.51, F(1, 214) = 115.1, *p* < 0.001: APS discrepancy was the most robust predictor of low PWB (β = −0.68, *p* < 0.001), followed by high standards with a positive direction (β = 0.23, *p* < 0.001). 

### 3.4. Mediational Model

**Figure 1:**
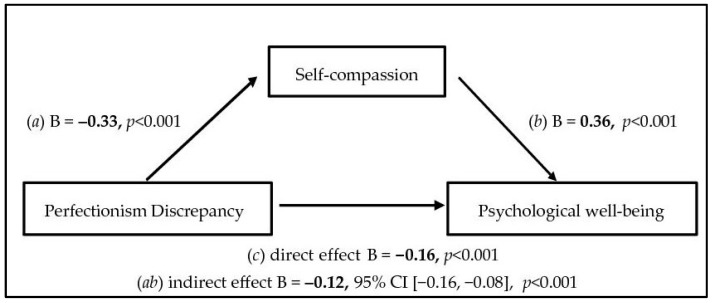
Direct and indirect effects for the relationship between unhealthy perfectionism (discrepancy) and psychological well-being, as mediated by self-compassion.

The hypothesized mediation model assumed direct and indirect effects for the relationship between unhealthy perfectionism (discrepancy) and psychological well-being, as mediated by self-compassion. All paths are described in Figure 1. Higher scores on discrepancy had a significant direct effect on lower self-compassion scores (path a, discrepancy to self-compassion = −0.33, 95% CI [−0.38, −0.28], *p* < 0.001) and lower psychological well-being scores (path c, discrepancy on psychological well-being = −0.16, 95% CI [−0.21, −0.11], *p* < 0.001). Higher scores on self-compassion had a significant direct effect on higher psychological well-being (path b, self-compassion on psychological well-being = 0.36, 95% CI [0.26, 0.46], *p* < 0.001).

Bootstrapping indicated that the indirect path ab (linking discrepancy to psychological well-being via self-compassion) was = −0.12, 95% CI [−0.16, −0.08], *p* < 0.001. The Sobel test for the significance of the mediation effect was z = −6.02, *p* < 0.001. Therefore, this indirect effect suggests a reduction of the initial weight of discrepancy on psychological well-being levels.

Finally, the total effect (i.e., combined direct and indirect effects) of discrepancy on psychological well-being was −0.28, 95% CI [−0.32, −0.24], *p* < 0.001.

## 4. Discussion

The long tradition of studies on the influence of perfectionism on the onset of psychopathology [14,42] probably overshadowed interest in healthy outcomes. This has occurred despite the intuition of Stroeber and Otto [8], who had argued for the potential positive consequences of perfectionism traits, such as subjective happiness, self-efficacy, or effective coping [9,16,17,19]. As far as authors know, few studies have explored the link between adaptive and maladaptive perfectionism and psychological well-being, with some exceptions (for example, [43,44]). Therefore, this study focused on the role of multidimensional perfectionism in psychological well-being. In addition, this study assumed an eudaimonic perspective of well-being [28] that connects subjective well-being (hedonic perspective, i.e., happiness or life satisfaction) with the individual’s sense of full self-realization and involvement in life circumstances. Particularly, this composite model of psychological well-being includes the personal perception of competence and growth over time with experiences that meet the individual’s values and direction of life. This eudaimonic perspective is recognized as relevant for adults’ perspectives on life [40,45].

First, maladaptive perfectionism intended as discrepancy with respect to the expected standard resulted in the most robust predictor of low psychological well-being. These findings are in line with existing literature that evidenced how maladaptive perfectionism leads people to accentuate their imperfections, experimenting feelings of inadequacy, anxiety, and distress [46,47]. Conversely, high standards reflect the positive face of perfectionism, since people set realistic goals, take self-oriented actions, and strive to do their best, experimenting the satisfaction of accomplishment [48]. The results of the current study show positive associations between elevated standards and higher well-being, particularly the dimensions of purpose in life, environmental mastery, self-acceptance, and personal growth of Ryff’s scale. Findings extend to adults what was already observed among undergraduate students. Hill and colleagues [31] found that perfectionistic strivings, conceived as a healthy face of perfectionism, were positively linked with purpose in life. Gaudreau and Thompson [49] observed that healthy perfectionists—characterized by high academic standards and low evaluative concerns—report higher well-being than students who are constantly concerned about their performance. Finally, in Park and Jeong’s [30] study, adaptive perfectionists obtained higher scores on purpose in life and personal growth than maladaptive perfectionists. As some scholars underlie [45], self-acceptance and personal growth, along with purpose in life and environmental mastery, can be conceived as “health assets” of adults’ functioning. Particularly in the transition to adulthood, people may face growing challenges in various social and environmental situations, but they still want to maintain their autonomy and direction in life. The acceptance of their own limitations, without self-criticism and fear of social judgment, can help people work on personal projects, focusing more on perseverance and self-oriented behaviors than uniquely on performance results [50]. 

In this study, self-compassion was linked with higher psychological well-being, as originally proposed by Neff [51] and confirmed in the literature, e.g., [52]. More particularly, results show positive associations between the key dimensions of self-compassion (self-kindness, common humanity, and mindfulness) and eudaimonic well-being. Self-compassionate participants show a more realistic perceptions of themselves and tolerance for one’s limits (self-acceptance), a sense of continuous development (personal growth) and meaning for one’s existence (purpose in life), satisfaction with relationships (positive relations), self-determination (autonomy), and the ability to actively manage the circumstances (environmental mastery). Self-compassion has been linked with a variety of positive factors related to well-being, such as optimism, happiness, self-esteem, social cohesion, emotional intelligence, openness, and cognitive flexibility, among others (see [24,51] for a review). Particularly, research suggests that self-compassionate individuals are more likely to focus on mastery goals than on results [16,44]; furthermore, they exhibit more effective coping (such as cognitive reappraisal, acceptance, and problem-solving) and less behavioral avoidance, catastrophic thinking, and emotion suppression than people who are not compassionate toward themselves [53]. Similarly, self-compassion seems to intervene in the mechanisms of emotions regulation, reducing negative reactions (e.g., rumination, self-devaluing thoughts) to difficult experiences. Moreover, self-compassionate people tend to tolerate unpleasant situations, accepting their responsibilities without feeling overwhelmed with negative emotions [54]. Consequently, people maintain a positive attitude toward themselves, and they seem less vulnerable to stress, fear of failure, and depressive symptoms [35]. Treating oneself with compassion may be a resource when people experience challenges and failures, since it encourages action to change and supports personal growth and well-being [51].

As for the dimensions of perfectionism, no relationship emerged between adaptive perfectionism (personal standards and order) and self-compassion. These findings are in accordance with Neff [51] and also replicate results from Ferrari and colleagues [26]. In line with the hypothesis and previous studies (e.g., [25]), strong negative associations were found between discrepancy and self-compassion sub-scales. The discrepancy is characterized by excessive self-criticism, dissatisfaction, and perceived distance between expectations and actual levels of performance. In other words, maladaptive perfectionists tend to set goals that are unrealistic, and, above all, they perceive incongruence between ideal standards and actual performance; in turn, self-criticism as a reaction to imperfections or mistakes may negatively impact self-esteem [16,21] and be associated with distress, shame, a feeling of inadequacy, and depressive symptoms [5,25,48]. This excessive self-criticism is the opposite of self-compassion, that is, being tolerant of one’s flaws and inadequacies. People with high levels of maladaptive perfectionism and low self-compassion in the long-term show general distress, anxiety, and depressive symptoms [27]. Conversely, self-compassion positively predicted subjective well-being (satisfaction with life) and negatively predicted depression and negative affect [23]. Consequently, it is important to consider to what extent self-compassion can mitigate the impact of maladaptive perfectionism.

The mediational analysis confirms that self-compassion significantly mediates the association between discrepancy and low psychological well-being, reducing the impact of maladaptive perfectionism on psychological well-being. Previous studies found that self-compassion attenuates self-criticism [26,34] and the negative reactions (such as a sense of failure and humiliation) to unpleasant life events [54], alleviating depressive and anxiety symptoms in emerging adults [35]. In the current study, a focus on positive psychological functioning was posed, and findings support the hypothesis on the role of self-compassion in alleviating the negative impact of discrepancy on well-being levels. As Neff suggests [24], self-compassion is a healthy attitude that can soften self-criticism through various mechanisms. Self-compassion can help perfectionistic people recognize their imperfections for what they are, that is, considering the failures as part of human experience (common humanity) and not a personal condition of which to be ashamed (“it only happens to me”). Mindfulness can facilitate people to be aware of their internal states (“I thought I could do it, but it’s hard”), including the severe self-judgments and the suffering they cause (e.g., “I’m not perfect, it’s my fault if I don’t succeed” or “People will judge me badly”). Be aware of these internal states, particularly recognizing the hypercritical judgment towards oneself, which allows people to change their attitude and treat themselves with benevolence and understanding (e.g., being kind to yourself even when you cannot reach your best). Therefore, self-compassion is a psychological resource that should be cultivated, especially in individuals with high levels of maladaptive perfectionism [35,55].

This study has certain limitations that warrant consideration. First, the convenience sample is small in size, and it comes from participants who voluntarily joined the research disseminated online. Therefore, the generalizability of the data is reduced, and future research enlarging the sample size is needed. Secondly, the sample was unbalanced by gender (more women), probably due to the more likely involvement of females in social media. However, this study offers an empirical contribution to data still lacking in the field of perfectionism and positive psychology among emerging adults. Whereas existing literature provides wide support for the role of self-compassion in decreasing the impact of maladaptive perfectionism on unhealthy outcomes (mainly, depressive symptoms [25,27,44]), this study has the merit of focusing on multidimensional perfectionism and well-being, thus offering insights for intervention. 

## 5. Conclusions

Psychological interventions based on self-compassion can be advantageous for promoting psychological well-being in individuals with perfectionist tendencies. Findings from the current study confirm the multifaced nature of perfectionism (according to Slaney et al. [12]), in which adaptive perfectionism (i.e., high personal standards) is linked to higher psychological well-being. Additionally, self-compassion resulted in a protective factor towards the psychological well-being of individuals with high levels of maladaptive perfectionism (i.e., discrepancy). These findings suggest that focusing on self-compassion is crucial for promoting healthy psychological functioning and reducing perfectionist thoughts. Existing cognitive-behavioral interventions (CBIs) often aim to directly modify maladaptive perfectionism thoughts, as they are recognized as a trans-diagnostic component of psychological distress and a predictive factor of depression [56]. CBIs encourage people to reappraise dysfunctional cognitions (e.g., “If I don’t reach my best, I’m a failure”), leading to a change in excessive evaluative concerns and negative feelings. School-based interventions can help the students recognize their beliefs about societal or parental pressures for excellence [57], reducing their vulnerability to maladaptive perfectionism (i.e., thinking that personal value or self-esteem does not depend on meeting external expectations). Furthermore, when students perceive teachers as supportive (i.e., teachers recognize student’s value as a person, regardless of achievement), their perfectionistic concerns decrease [58]. Alternatively, a complementary approach based on self-compassion helps people modify the relationship with their own thoughts of imperfection and failure, and to treat themselves with kindness [26]. An example of intervention based on self-compassion is a brief group course (2 weeks, 3 sessions) for university students by Binder and colleagues [59]. The sections introduced the core themes of mindfulness and self-compassion through a brief 15 min presentation, followed by group discussion and experiential exercises (e.g., how to become more friendly and supportive toward yourself, recognizing self-criticism and emotive reactions, etc.). Following these guided self-compassion practices, participants reported greater acceptance of painful emotions and a decrease in their habitual self-criticism [59]. James and Rimes [60] found that a group intervention focused on mindfulness-based cognitive therapy (MBCT) resulted in more efficacy in increasing self-compassion and reducing perfectionist thoughts, negative emotions, and distress than a traditional self-help CBI. Similarly, Woodfin and colleagues [61] demonstrated the beneficial outcomes of a brief (3-week) intervention based on self-compassion in a university setting. Participants followed group seminars, short lectures, and experiential practices based on mindfulness, self-compassion (e.g., how they behave with themselves and how they behave with others in unpleasant situations), and one section specifically dedicated to perfectionism and core values. Daily exercises were also suggested, and the support of a recorded audio guide was offered. Results indicate a significant increase in self-compassion levels and a reduction of maladaptive perfectionism and anxiety/depression symptoms in the intervention group compared to the control (waiting list) group. These interventions based on self-compassion appear to be a promising approach to enhancing resilience and mitigating perfectionistic tendencies that can damage individual well-being. 

## Figures and Tables

**Table 1:** Descriptive statistics (means and SD) and correlations (Pearson’s coefficients) among the study variables (N = 217).

Variable	M	SD	1	2	3	4	5	6	7	8	9	10	11	12	13	14	15
APS 1. High standards.	5.33	0.80	1														
2. Discrepancy	4.07	1.34	0.01	1													
3. Order	5.24	0.95	0.30 **	0.07	1												
SCS 4. Self-kindness	3.07	0.90	−0.05	−0.47 **	−0.09	1											
5. Self-judgment	2.90	0.94	−0.05	−0.63 **	0.00	0.13 *	1										
6. Common humanity	3.22	0.89	−0.03	−0.29 **	−0.07	0.61 **	0.03	1									
7. Isolation	2.89	1.06	0.10	−0.63 **	−0.03	0.15 **	0.71 **	0.08	1								
8. Mindfulness	3.28	0.86	0.10	−0.33 **	−0.13	0.67 **	0.12 *	0.56 **	0.17 **	1							
9. Over-identification	2.86	1.04	0.12	−0.66 **	−0.07	0.09	0.76 **	0.01	0.76 **	0.12 *	1						
PWS 10. Positive relations	4.14	0.90	0.06	−0.40 **	0.06	0.30 **	0.09	0.23 **	0.09	0.22 **	0.10	1					
11. Self-acceptance	3.90	1.00	0.18 **	−0.69 **	0.03	0.63 **	0.16 **	0.49 **	0.12 *	0.53 **	0.14 *	0.47 **	1				
12. Purpose in life	4.15	0.94	0.29 **	−0.63 **	0.05	0.46 **	0.17 **	0.36 **	0.17 **	0.45 **	0.18 **	0.53 **	0.82 **	1			
13. Autonomy	4.28	0.80	0.11	−0.37 **	0.01	0.28 **	0.03	0.24 **	0.04	0.28 **	0.03	0.34 **	0.46 **	0.44 **	1		
14. Environmental	3.91	0.60	0.29 **	−0.53 **	0.19 **	0.46 **	0.11 *	0.36 **	0.08	0.42 **	0.10	0.45 **	0.74 **	0.70 **	0.45 **	1	
15. Personal growth	4.78	0.70	17*	−0.41 **	0.04	0.37 **	0.05	0.40 **	0.02	0.32 **	0.03	0.50 **	0.60 **	0.60 **	0.52 **	0.55 **	1

Note: APS-R = Almost Perfect Scale-Revised; SCS = Self-Compassion Scale; PWS = Psychological Well-being Scale; M = mean; SD = standard deviation. Correlation significant at * 0.05 or ** 0.01 level (two-tailed).

**Table 2:** Summary of regression analysis for Perfectionism dimensions (APS-R) predicting overall PWB.

Model		Unstandardized Coefficients		Standardized Coefficients			95% Confidence Interval for B	
		B	SE	β	t	Sig.	Lower Bound	Upper Bound
1	(Constant)	5.24	0.09		59.66	<0.001	5.06	5.41
	ALMOST—Discrepancy	−0.28	0.02	−0.68	−13.64	<0.001	−0.32	−0.24
2	(Constant)	4.39	0.19		22.99	<0.001	4.01	4.77
	ALMOST—Discrepancy	−0.28	0.02	−0.68	−14.42	<0.001	−0.32	−0.24
	ALMOST—High Standard	0.16	0.03	0.23	4.92	<0.001	0.10	0.22

Note: N = 217, SE = standard error.

## Data Availability

The data presented in this study are available on request from the corresponding author due to legal restrictions.
